# Supplementary material for: Associations between Vitamin D, Omega 6:Omega 3 Ratio, and Biomarkers of Aging in Individuals Living with and without Chronic Pain
Source: Nutrients. 2022 Jan 9;14(2):266. doi: 10.3390/nu14020266 (PMC8779718; doi:10.3390/nu14020266)
Supplement: Supplementary file 1 [file nutrients-14-00266-s001.zip › nutrients-1532941-supplementary.pdf]

*Supplementary Materials*

**Associations between Vitamin D, Omega 6: Omega 3 Ratio, and Biomarkers of Aging in Individuals Living with and without Chronic Pain**

Akemi T. Wijayabahu [1,2], Angela M. Mickle [3,4], Volker Mai [1,2], Cynthia Garvan [5], Toni L. Glover [6], Robert L. Cook [1,2], Jinying Zhao [1], Marianna K. Baum [7], Roger B. Fillingim [4], and Kimberly T. Sibille [3,4,5,*]

Supplemental tables S1-S4 are presented in this section.

**Table S1.** Associations between selected serum micronutrient categories and leukocyte telomere length in people living with and without chronic pain

| | | **Unadjusted** | | **Adjusted** | |
|---|---|---|---|---|---|
| | | **N** | **β (95% CI)** | **N** | **β (95% CI)** |
| Vitamin D | | | | | |
| | Deficiency | 80 | 0.02 (-0.05, 0.09) | 79 | -0.04 (-0.12, 0.04) |
| | Insufficiency | 75 | 0.02 (-0.05, 0.09) | 74 | <0.01 (-0.07, 0.07) |
| | Sufficiency | 54 | Reference | 53 | Reference |
| Omega 6:3 Ratio | | | | | |
| | >Recommended | 115 | <-0.01 (-0.13, 0.12) | 114 | 0.03 (-0.08, 0.1) |
| | ≤Recommended | 12 | Reference | 12 | Reference |
| Combined model | | | | | |
| Vitamin D | | | | | |
| | Deficiency | 45 | 0.03 (-0.06, 0.12) | 44 | -0.05 (-0.15, 0.04) |
| | Insufficiency | 45 | 0.05 (-0.05, 0.14) | 45 | 0.02 (-0.07, 0.11) |
| | Sufficiency | 37 | Reference | 37 | Reference |
| Omega 6:3 Ratio | | | | | |
| | >Recommended | 115 | -0.01 (-0.13, 0.11) | 114 | 0.02 (-0.09, 0.13) |
| | ≤Recommended | 12 | Reference | 12 | Reference |

Abbreviations: CI (Confidence Interval), LTL (Leukocyte Telomere Length), Omega 6: Omega 3 ratio (Omega 6:3 ratio), Waist Hip Ratio (WHR)

Regression estimates rounded to their nearest hundredth and < 0.01 assigned for lower values. Multivariable model adjustments: age, sex, race, study site, WHR, physical activity and number of pain sites. ; LTL was log transformed. Variable categories: serum vitamin D (deficiency: <20ng/mL, insufficiency 20-30ng/mL, sufficiency >30ng/mL) [51], omega 6:3 ratio (upper limit cut-off value for recommended intake: 5) [47, 52].

Combined model: regression models included both vitamin D and omega 6:3 ratio as predictors along with other covariates

**Table S2.** Associations between selected serum micronutrient categories and C-reactive proteins in people living with and without chronic pain

| | | Unadjusted | | Adjusted | |
|---|---|---|---|---|---|
| | | N | β (95% CI) | N | β (95% CI) |
| Vitamin D | | | | | |
| | Deficiency | 69 | **1.00 (0.57, 1.44)** | 67 | **0.56 (0.10, 1.02)** |
| | Insufficiency | 88 | 0.11 (-0.32, 0.53) | 86 | -0.12 (-0.54, 0.31) |
| | Sufficiency | 54 | Reference | 50 | Reference |
| Omega 6:3 Ratio | | | | | |
| | >Recommended | 147 | **0.83 (0.16, 1.50)** | 141 | **0.75 (0.12, 1.38)** |
| | ≤Recommended | 16 | Reference | 16 | Reference |
| Combined model | | | | | |
| Vitamin D | | | | | |
| | Deficiency | 51 | **0.80 (0.29, 1.30)** | 49 | 0.44 (-0.09, 0.97) |
| | Insufficiency | 68 | -0.16 (-0.64, 0.31) | 66 | -0.30 (-0.79, 0.19) |
| | Sufficiency | 44 | Reference | 42 | Reference |
| Omega 6:3 Ratio | | | | | |
| | >Recommended | 147 | **0.74 (0.09, 1.39)** | 141 | **0.76 (0.15, 1.37)** |
| | ≤Recommended | 16 | Reference | 16 | Reference |

Abbreviations: CRP (C-Reactive Protein), CI (Confidence Interval), Omega 6: Omega 3 ratio (Omega 6:3 ratio), Waist Hip Ratio (WHR)

Multivariable model adjustments: age, sex, WHR, physical activity, smoking status, number of comorbidities, annual household income and number of pain sites. ; CRP was log transformed. Variable categories: serum vitamin D (deficiency: <20ng/mL, insufficiency 20-30ng/mL, sufficiency >30ng/mL) [51], omega 6:3 ratio (upper limit cut-off value for recommended intake: 5) [47, 52]. Combined model: regression models included both vitamin D and omega 6:3 ratio as predictors along with other covariates

**Table S3.** Associations between continuous micronutrients and C-reactive protein stratified by pain status (adjusted models)

| | Overall | | Chronic Pain | | No Chronic Pain | |
|---|---|---|---|---|---|---|
| | N | β (95% CI) | N | β (95% CI) | N | β (95% CI) |
| Vitamin D | 203 | -0.02 (-0.04, <0.01) | 163 | **-0.02 (-0.05, <-0.01)** | 40 | 0.01 (-0.02, 0.05) |
| Omega 6:3 Ratio | 157 | **0.11 (0.01, 0.21)** | 124 | **0.15 (0.02, 0.28)** | 33 | -0.05 (-0.35, 0.25) |
| Combined model | | | | | | |
| Vitamin D | 157 | -0.01 (-0.03, 0.01) | 124 | -0.02 (-0.05, 0.01) | 33 | 0.04 (-0.01, 0.09) |
| Omega 6:3 Ratio | 157 | 0.10 (<-0.01, 0.21) | 124 | **0.14 (0.01, 0.27)** | 33 | 0.04 (-0.27, 0.34) |

Abbreviations: CRP (C-Reactive Protein), CI (Confidence Interval), Omega 6:3 Ratio (Omega 6: Omega 3 Ratio), Waist Hip Ratio (WHR)

Regression estimates rounded to their nearest hundredth and < 0.01 assigned for lower values. Multivariable model adjustments: age, sex, WHR, physical activity, smoking status, number of comorbidities, annual household income and number of pain sites; CRP was log transformed. Combined model: regression models included both vitamin D and omega 6:3 ratio as predictors along with other covariates. Significant associations after adjustment with NSAIDs in people living with pain: vitamin D (β -0.02, 95% CI -0.05, -0.001)

**Table S4.** Associations between categorical micronutrients and C-reactive proteins in people living chronic pain

| | Adjusted | | Adjusted models with NSAIDs | |
|---|---|---|---|---|
| | N | β (95% CI) | N | β (95% CI) |
| Vitamin D | | | | |
| Deficiency | 56 | **0.69 (0.14, 1.24)** | 56 | **0.68 (0.13, 1.22)** |
| Insufficiency | 68 | 0.01 (-0.05, 0.51) | 68 | 0.02 (-0.49, 0.53) |
| Sufficiency | 39 | Reference | 39 | Reference |
| Omega 6:3 Ratio | | | | |
| >Recommended | 112 | **0.89 (0.13, 1.65)** | 112 | **0.83 (0.08, 1.59)** |
| ≤Recommended | 12 | Reference | 12 | Reference |
| | | | | |
| Combined model | | | | |
| Vitamin D | | | | |
| Deficiency | 40 | 0.60 (-0.05, 1.24) | 49 | **0.64 (0.01, 1.28)** |
| Insufficiency | 50 | -0.30 (-0.89, 0.28) | 66 | -0.31 (-0.90, 0.28) |
| Sufficiency | 34 | Reference | 42 | Reference |
| Omega 6:3 Ratio | | | | |
| >Recommended | 112 | **0.86 (0.12, 1.59)** | 141 | **0.78 (0.06, 1.50)** |
| ≤Recommended | 12 | Reference | 16 | Reference |

Abbreviations: CRP (C-Reactive Protein), CI (Confidence Interval), Omega 6: Omega 3 Ratio (Omega 6:3 Ratio) Multivariable model adjustments: age, sex, WHR, physical activity, smoking status, number of comorbidities, annual household income and number of pain sites. ; CRP was log transformed. Variable categories: serum vitamin D (deficiency: <20ng/mL, insufficiency 20-30ng/mL, sufficiency >30ng/mL) [51], omega 6:3 ratio (upper limit cut-off value for recommended intake: 5) [47, 52]. Combined model: regression models included both vitamin D and omega 6:3 ratio as predictors along with other covariates
